# Anthocyanin Profile of Elderberry Juice: A Natural-Based Bioactive Colouring Ingredient with Potential Food Application

**DOI:** 10.3390/molecules24132359

**Published:** 2019-06-26

**Authors:** Ricardo F. R. da Silva, João C. M. Barreira, Sandrina A. Heleno, Lillian Barros, Ricardo C. Calhelha, Isabel C. F. R. Ferreira

**Affiliations:** Centro de Investigação de Montanha (CIMO), Instituto Politécnico de Bragança, Campus de Santa Apolónia, 5300-253 Bragança, Portugal

**Keywords:** *Sambucus nigra* L., anthocyanins, natural colourant, bioactivity, functionalised foods

## Abstract

Elderberry (*Sambucus nigra* L.) is a widely disseminated plant that produces bright black berries containing high quantities of anthocyanins, acknowledged for their bioactivity and dye capacity. Besides other applications, anthocyanins might be employed as natural colouring agents to reduce/eliminate the use of artificial dyes, while providing positive effects on consumers’ health. Herein, the anthocyanins profile of elderberry juice was characterised by HPLC-DAD-ESI/MSn. In addition, its antioxidant capacity and cytotoxicity were also evaluated. As a proof-of-concept of its colouring capacity, elderberry juice was added (different percentages) to a highly appreciated and consumed pastry product (croissant) and compared with a commercial dye (black carrot). In general, the nutritional properties of control and coloured croissants were similar, despite some individual differences in sugars and fatty acids. In turn, the appearance obtained with elderberry juice incorporation might be considered innovative, besides partially maintaining the anthocyanins content of fresh juice and showing considerable antioxidant activity.

## 1. Introduction

In line with current consumers’ demands, the food industry has been improving the functional quality of its products, while developing more appealing foodstuffs, since appearance (e.g., its colour) plays an important role when consumers select a specific food product [1]. However, the conventional employment of artificial colourants is starting to be severely unwanted, as these compounds are often associated with side effects such as allergic reactions, toxicity, and hyperactivity in children, among others [2,3]. Likewise, consumers are progressively pressuring food industry to provide new food products with the minimum artificial additives possible. As a consequence, the European Food and Safety Authority and the Food and Drug Administration has restricted the use of some colourants in foods, thereby increasing the need for effective alternatives [4,5]. In this context, natural matrices have been extensively explored to obtain natural colourants that are safe, non-toxic and, most of all, can act as health promoters due to their richness in specific bioactive molecules [5,6]. Anthocyanins are good examples of such doubly functional agents; the employment of these phenolic compounds in different food products has been previously validated, being classified as colouring agents with the code E163 [1]. One of the plant species with highest richness in anthocyanins is *Sambucus nigra* L., commonly known as elderberry. This shrub species grows preferentially in sunlight-exposed places in Europe, Asia, North Africa and USA. It can reach up to 6 m in height, and develops small white hermaphrodite flowers that blossom in early summer. The umbels consist of dark purple individual berries that reach full ripeness in late summer. *S. nigra* berries present good levels of proteins, free and conjugated forms of amino acids, unsaturated fatty acids, fibre, vitamins and minerals, flavonols, phenolic acids and proanthocyanidins, as well as terpenes and lectins [7,8,9]. With respect to their anthocyanin profile, these berries are especially rich in cyanidin-3-*O*-sambubioside (the major anthocyanin), cyanidin-3-*O*-sambubioside-5-*O*-glucoside, cyanidin-3,5-*O*-diglucoside, cyanidin-3-*O*-glucoside, and cyanidin-3-*O*-rutinoside [7,9]. However, this class of compounds presents some stability issues, since temperature, light, pH, enzymes, light, or oxygen, can influence their colouring capacity [10], demanding further studies before being applied as colouring and bioactive ingredients in novel food products.

The inclusion of bioactive ingredients in food products is less important in the case of diets rich in fruits and vegetables, such as the Mediterranean diet. However, the current lifestyle observed in most countries does not comply with a balanced diet, resulting in a demand for food functionalisation strategies that might counteract the lack of specific ingredients; these strategies may be particularly effective if practiced with popular foods, such as well exemplified by pastry products [11].

In this work, we’ve used elderberry juice to functionalise croissants, owing to the high consumption rates of this product worldwide [12], as also its relevant levels of protein and fibre [13,14].

The main objective of the present study was to characterise the anthocyanin profile of elderberry juice and further evaluating its bioactivity. This upstream study allowed to define suitable concentrations of juice to functionalise croissants, developing a product with an innovative appearance and potentially improved health effects. The final products were thoroughly analysed in terms of nutritional value, chemical composition and anthocyanins profile.

## 2. Results and Discussion

### 2.1. Bioactivity and Anthocyanin Profile of S. nigra Juice

Fresh elderberry juice (after straining) was lyophilised, and further reconstituted in deionised water. The solid content of juice was determined as being 9.375 g/100 g. This solid fraction was further submitted to successive dilutions, setting a range of concentrations varying from 9.375 μg/mL and 93.75 mg/mL (this last corresponding to the natural solid to liquid ratio of the juice).

Prior to its incorporation into croissants, elderberry juice was characterised for its bioactivity (particularly antioxidant activity and cytotoxicity) and anthocyanin profile.

With regard to its antioxidant activity (Table 1), the best results were obtained in DPPH scavenging activity (EC_50_ = 3.1 ± 0.1 mg/mL) and reducing power (EC_50_ = 3.7 ± 0.2 mg/mL), being less effective against β-carotene bleaching inhibition (EC_50_ = 9.4 ± 0.3 mg/mL).

In addition to the good antioxidant activity results, elderberry juice also showed significant capacity to inhibit the growth of different tumour cell lines. HeLa and NCI-H460 resulted in be the most sensitive, as indicated by the GI_50_ value (16 ± 1 μg/mL), while MCF-7 (GI_50_ = 58 ± 1 μg/mL) and HepG2 (GI_50_ = 98 ± 4 μg/mL) demonstrated higher resistance.

**Figure 1:**
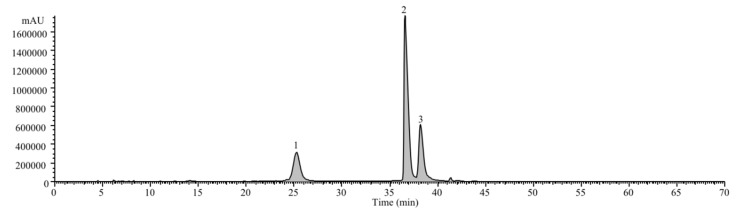
Chromatogram of identified anthocyanins in *Sambucus nigra*. 1: cyanidin-3-*O*-sambubioside-5-*O*-glucoside; 2: cyanidin-3-*O*-sambubioside; 3: cyanidin-3-*O*-glucoside.

The observed bioactivity should be, at least partially, justified by the profiled anthocyanins (Figure 1), which were all identified as having been derived from cyanidin, known for its positive health effects [15]. The most abundant (Table 1) was cyanidin-3-*O*-sambubioside (560 ± 45 μg/mL), followed by cyanindin-3-*O*-glucoside (390 ± 30 μg/mL) and cyanidin-3-*O*-sambubioside-5-*O*-glucoside (140 ± 21 μg/mL), which is in agreement with previous studies [7,9].

Cyanidin-3-*O*-glucoside was confirmed by comparison with a commercial standard, while the other anthocyanins were identified by comparison of the chromatographic and spectral characteristics (UV and mass) with data from our library of compounds and related literature [16,17].

### 2.2. Nutritional and Chemical Characterisation of Croissants

**Figure 2:**
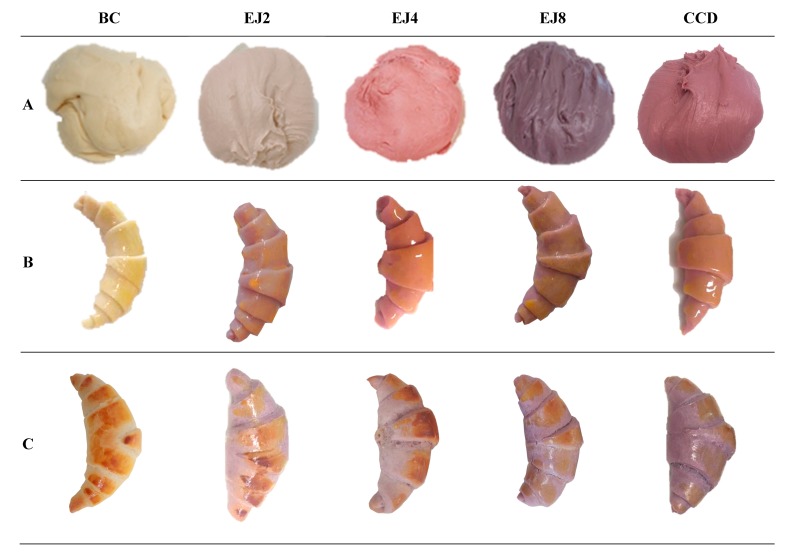
Appearance of different croissant formulations. (**A**) after second fermentation of batter; (**B**) before baking; (**C**) after baking. BC: croissant with no colorants adding; EJ2: croissant with 2% of elderberry juice (EJ2); EJ4: croissant with 4% of elderberry juice; EJ8: croissant with 8% of elderberry juice; CCD: croissant with 4% of the commercial carrot dye.

The daily recommended dietary dose for anthocyanins is not accurately defined, but there are some indications that it is about 50 mg per day [18]. Thus, when defining the percentages of elderberry juice to be added to croissants, in addition to the effect on the visual appearance, that maximum limit (although not validated) was considered. The highest tested percentage was 8% juice (32 g of juice in a total batter mass of 400 g), which corresponds to a maximum value of 34.88 mg (considering that 1 g of juice corresponds to approximately 1 mL) of anthocyanins (according to the values described in Section 3.1) per 400 g of croissant batter, or 8.72 mg per 100 g of croissant. Accordingly, even for an unlikely consumption of 500 g of croissant per day, the quantity of anthocyanins would still be inferior to the indicated threshold value (especially considering that a significant part of total anthocyanins might be lost in the baking process). The visual appearance of all prepared formulations might be observed in Figure 2, either before cooking, as well as after being baked. Besides measuring the colour parameters (L*, a* and b*), these different formulations were also compared for nutritional composition, free sugars profile, and fatty acids profile. To assess the effectiveness of extracts incorporation, the croissants were submitted to the extraction process described in Section 3.4 to quantify their anthocyanin content.

Differences among different croissant recipes were more evident when comparing individual molecules. In what concerns free sugars, the same quantitative ratio (fructose > maltose > glucose > trehalose) was found in all cases (Table 2). However, by comparing each free sugar individually, some significant differences were observed: fructose showed lower values in BC (4.1 g/100 g) and CCD (4.2 g/100 g); the highest maltose levels were detected in BC (3.9 g/100 g) and EJ8 (4.0 g/100 g), this last also presenting the highest glucose content (3.4 g/100 g); finally, trehalose presented the maximum value of BC (1.8 g/100 g). Independently of individual differences, it seems obvious that most carbohydrates are present as starch, since this group of macronutrients represents 70% of the mass of croissants, but the total free sugars correspond only to about 12%.

Table 2 shows the average nutritional values, in g/100 g of croissant, and the corresponding energy value, in kcal/100 g. Considering that the specific difference among formulations was the incorporation of small percentages of elderberry juice, it was not expectable to have significant differences in most nutritional parameters, as it was observed in the case of moisture and carbohydrates. Proteins and fat, in turn, showed minor (yet significant) differences; protein content was lower in BC and EJ2 (10.3 and 10.4 g/100 g, respectively), while fat content reached maximum values in BC (17.0 g/100 g), presenting the minimal value in EJ8 (16.1 g/100 g). Regarding energy values, a slight tendency for lower values in croissants with higher juice percentages was observed. Water content was only residual, certainly as a result of the baking process (180 °C). In general, the nutritional profile is in agreement with the published results [19].

In any case, it can be concluded that elderberry juice and black carrot dye did not affect the profile in free sugars in a relevant way, which is always a positive point in relation to the maintenance of the organoleptic characteristics of this product.

Among lipophilic compounds, fatty acids profiles are of utmost importance in this kind of food products. All major forms (relative abundance greater than 1%) are listed in Table 3 (the additional quantified fatty acids were C8:0, C11:0, C13:0 C18:3n6, C18:3n3, C20:0, C20:1, C20:2, C20:3n6, C20:3n3, C20:5n3, C22:0, C22:6n3 and C24:0, which, nonetheless, were included in the aggregated values of SFA, MUFA and PUFA).

Owing the hydrophilic nature of the incorporated juice, it was not expectable to have great differences in fatty acids profile. Nevertheless, all major forms presented significant differences (despite the overall similarity of values) for at least one croissant formulation. EJ8 showed high percentages of C6:0 (1.6%), C10:0 (1.9%), C12:0 (2.5%), C14:0 (7.6%), C16:0 (16%), and C18:0 (8.3%, in this case ex aequo with CCD); oleic acid presented the highest percentage in EJ4 (30%), similar to the observed for linoleic acid in EJ2 (29%) and BC (28%) and linolenic acid in EJ2 and EJ4 (1.3% in both cases). In both cases, the fact that all croissants presented approximately 60% of unsaturated fatty acids is a relevant nutritional advantage, considering their recognised beneficial effects [20].

### 2.3. Colour Parameters

Considering that one of the main objectives of the work was obtaining a new croissant formulation with an innovative appearance, the colour parameters (Table 4), namely, L*, a*, and b*, were evaluated.

In samples prepared with elderberry juice, the increase in juice concentration caused a reduction in lightness (L* = 54, in EJ8), which in turn presented the highest value in CB (L* = 79). This particular change might be related to the darker colour of croissants added with elderberry juice (Figure 2), which, on the other hand, showed higher redness (a*) values (13.9 in EJ4 and 14.0 in EJ8). Regarding this last parameter, an important observation is related to the absence of a significant difference among EJ4 and EJ8, indicating that the addition of 4% elderberry juice would be the best choice, since the same appearance change could be obtained with half of the juice, when compared to EJ8. With regard to yellowness (b*), the highest values were obtained in CC (b* = 31), while the lowest were measured in EJ8 (b* = 9).

### 2.4. Antioxidant Activity and Anthocyanin Content of Prepared Croissants

To verify if the antioxidant activity found in elderberry juice was maintained in the prepared croissants, extracts (prepared as described in Section 3.8) from EJ4 (selected according to the results in Section 2.1) were evaluated.

According to the values presented in Table 1, and if no activity has been lost, the expected EC_50_ values would be 3.1 mg/mL for DPPH scavenging activity, 3.7 mg/mL for reducing power and 9.4 mg/mL for β-carotene bleaching inhibition. However, the determined values were significantly higher (8.6 mg/mL for DPPH scavenging activity, 10.2 mg/mL for reducing power and 30.5 mg/mL for β-carotene bleaching inhibition), which might be explained by some bioactive compound degradation during the baking process and/or a higher quantity of impurities in the extracts obtained from croissants. Nonetheless, the addition of elderberry juice is advantageous, since CB did not show any activity when submitted to the same tests. The lack of antioxidant activity in CB also indicates that the measured activity should be due to the anthocyanins in elderberry juice, and not to the melanoidines potentially formed through Maillard reactions in the baking process [21].

In addition to antioxidant activity evaluation, the anthocyanin content of EJ4 was also quantified to assess their stability to the baking process. The extraction procedure was applied as described in Section 3.4. For comparison purposes, the stability of anthocyanins present in CCD was also evaluated. To simplify the comparison, only the major anthocyanins in each case, i.e., cyanidin-3-*O*-sambubioside in elderberry juice and cyanidin-3-*O*-xylosyl(feruloylglucosyl)galactoside in commercial black carrot dye were analysed. After performing a second set of chromatographic analyses (as described in Section 3.4), it was verified that cyanidin-3-*O*-sambubioside in EJ4 and cyanidin-3-*O*-xylosyl(feruloylglucosyl)galactoside in CCD could be quantified in levels corresponding to approximately 45% and 48% of those found in elderberry juice and in the commercial black carrot dye. In similar works, related decreases in anthocyanin content were also observed, namely in pasta [11]. This represents a relevant reduction, which might be justified by the high temperature employed in the baking process; nevertheless, it can be concluded that elderberry juice acted as a dye and a functionalising agent, which undoubtedly adds quality to the confectionery product that was intended to be improved.

## 3. Experimental Section

### 3.1. Standards and Reagents

Acetonitrile and methanol were of HPLC grade and purchased from Fisher Scientific (Lisbon, Portugal). Trolox (6-hydroxy-2,5,7,8-tetramethylchroman-2-carboxylic acid), β-carotene, ellipticine, acetic acid, phosphate buffered saline (PBS), sulforhodamine B (SRB), trypan blue, Tris-(hydroxymethyl)aminomethan (TRIS), trichloroacetic acid (TCA), and lipopolysaccharide (LPS) were acquired from Sigma-Aldrich (St. Louis, MO, USA). Anthocyanin standards were from Extrasynthèse (Genay, France). DPPH (2,2-diphenyl-1-picrylhydrazyl) was obtained from Alfa Aesar (Ward Hill, MA, USA). Dulbecco’s Modified Eagle’s Medium (DMEM) and RPMI-1640 medium, fetal bovine serum (FBS), Hank’s balanced salt solution (HBSS), L-glutamine, nonessential amino acid solution (2 mM), penicillin/streptomycin solution (100 U/mL and 100 mg/mL, respectively), and trypsin-EDTA (ethylenediaminetetraacetic acid) were from Hyclone (Logan, UT, USA). Water was treated in a Milli-Q water purification system (TGI Pure Water Systems, Greenville, SC, USA) before use. All other chemicals and solvents were of analytical grade and purchased from common suppliers.

### 3.2. Sample Collection and Preparation

Berries from spontaneous *S. nigra* shrubs integrated in the local riparian flora were collected in September, 2016, according to the colouration (bright black) corresponding to complete maturation. Umbels were harvested from several representative plants; afterwards, berries were carefully separated from stalks, and the juice (approximately a total of 2 L) was obtained by mechanical pressure (without crushing the seeds) using a pestle and a strainer. The filtered juice was immediately frozen and lyophilised until further analysis.

### 3.3. Bioactive Properties

#### 3.3.1. Antioxidant Activity

Lyophilised elderberry juice was reconstituted with deionised water; samples were successively diluted in water to produce a concentration ranging from 100% to 0.1%, filtered (Whatman nº4) and immediately analysed using three in vitro antioxidant assays: DPPH radical-scavenging, reducing power (RP), and inhibition of β-carotene bleaching in the presence of linoleic acid radicals [22]. Results were expressed as EC_50_ values (concentrations providing 50% of inhibition in the case of DPPH and β-carotene bleaching inhibition, or 0.5 of absorbance for reducing power assay) in mg/mL. Trolox was used as positive control.

#### 3.3.2. Cytotoxicity

Juice samples were lyophilised (Büchi R-20, Flawil, Switzerland), diluted in water at 8 mg/mL, and successively diluted to test a range of concentrations (400 to 0.125 µg/mL). Cytotoxicity was evaluated using four cell lines: human breast adenocarcinoma (MCF-7), human non-small lung carcinoma (NCI-H460), human cervical carcinoma (HeLa), and human hepatocellular carcinoma (HepG2). A non-tumour primary culture, obtained from porcine liver cells (PLP2), was also used to analyse the cytotoxic effects in normal cells. The sulforhodamine B assay was used [23]. The results are expressed in GI_50_ values (concentrations providing 50% of cells inhibition; µg/mL). Ellipticine was used as positive control.

### 3.4. Anthocyanin Analysis

Lyophilised samples (∼1 g of elderberry juice or croissant) were extracted with methanol:water (80:20 *v*/*v*, 30 mL) containing 0.5% of trifluoroacetic acid (25 °C, 150 rpm, 1 h) and further filtrated (filter paper Whatman No.4). The extraction procedure was repeated over the residue remaining from the first extraction. The combined extracts were further concentrated under reduced pressure at 40 °C (rotary evaporator BüchiR-210, Flawil, Switzerland) and the remaining water lyophilised [24]. The obtained dried extracts were re-dissolved in water/methanol (80:20, *v*/*v*) at 10 mg/mL and filtered through a 0.22-μm disposable LC filter disk. Anthocyanins were analysed by high performance liquid chromatography coupled to a diode array detector and electrospray ionisation tandem mass spectrometry (HPLC-DAD-ESI/MSn; Dionex Ultimate 3000 UPLC, Thermo Scientific, San Jose, CA, USA). The chromatographic separation was achieved in an AQUA^®^ (Phenomenex) reverse phase C18 column (5 μm, 150 mm × 4.6 mm i.d). Anthocyanins were eluted with 0.1% trifluoroacetic acid in water (A) and acetonitrile (B) and further identified with double online detection mode using DAD (280, 330 and 370 nm, or 520 nm) coupled to a mass spectrometer equipped with an ESI source (Linear Ion Trap LTQ XL mass spectrometer; Thermo Finnigan, San Jose, CA, USA), working in positive mode [25]. Quantification was achieved with the calibration curve of the correspondent anthocyanin standard or, if not available, with the most similar compound. Results were recorded and processed using the Xcalibur data system (Thermo Finnigan, San Jose, CA, USA) and expressed in μg/mL.

### 3.5. Croissant Preparation

Croissants were prepared following a traditional recipe: flour (225 g), half-fat milk (80 mL), fresh yeast (5 g), egg, egg yolk, sugar (23.5 g), butter (32 g), sunflower oil (18 mL). In addition, filtered elderberry juice was added in different percentages, without exceeding the daily recommended maximum intake of anthocyanins (50 mg), according to the content determined chromatographically. Five formulations were prepared (percentages were calculated in *w*/*w* basis): (i) croissant with 2% of elderberry juice (EJ2); (ii) croissant with 4% of elderberry juice (EJ4); (iii) croissant with 8% of elderberry juice (EJ8); (iv) croissant with 4% of the commercial carrot dye (CCD); (v) croissant with no colourants added (BC). Commercial black carrot dye was generously provided by CHR Hansen, Hoersholm, Denmark; before incorporation, the dye solution was successively diluted until obtaining the absorbance value of elderberry juice at 4% concentration.

After batter preparation in an electric mixer (SilverCrest, SKMP 1300 B3), it was allowed to ferment for 1 h (37 °C). Dough was then kneaded and stretched into rectangles of 10 × 50 × 0.5 cm, from which the triangular sections for the croissants were cut. A second fermentation was left to occur, maintaining the same conditions employed in the first fermentation. All croissants were brushed with egg yolk to obtain a glazing effect, placed in a paper tray and baked in a preheated oven at 180 °C for 20 min. Afterwards, 9 croissants of each formulation were selected and further lyophilised to remove any residual water and finely ground (20 mesh) in order to facilitate their use in subsequent analysis.

### 3.6. Nutritional Value

Nutritional parameters (protein, fat, and ash; g/100 g of croissant.) were evaluated according to the AOAC methods [26]; the Jones factor utilised to calculate protein content was 5.83 (owing to the fact that wheat flour was the major ingredient). Carbohydrates were calculated as the difference among the sum of protein, fat, ash and water and 100 g of fresh product. Total energy was calculated following the equation:

Energy (Kcal) = 4 × (g_proteins_ + g_carbohydrates_) + 9 × (g_lipids_) (1)

Fatty acids were determined in hexane extracts (Soxhlet), after a methyl-esterification process, by gas chromatography coupled to a flame ionisation detector (GC-FID). The identification was carried out by comparison with standards (standard 47885, Sigma-Aldrich, St. Louis, MO, USA) and expressed as relative percentage of each fatty acid [27].

Individual sugars were characterised in hydro-ethanolic (80:20) extracts by HPLC coupled to a refraction index (RI) detector [27]. Sugar identification was performed by comparison with standards; quantification (g/100 g of croissant) was performed using the internal standard (melezitose) method.

### 3.7. Evaluation of Colour Changes

To validate the appearance change induced by incorporated colouring agents, the colour was measured on three different points of all croissants using a colorimeter (model CR-400, Konica Minolta Sensing Inc., Tokyo, Japan). The illuminate C was used, with a diaphragm aperture of 8 mm, after being calibrated against a standard white tile. The CIE L* (lightness), a* (greenness/redness), b* (blueness/yellowness) colour space values were registered using “Spectra Magic Nx” (version CM-S100W 2.03.0006) [27].

### 3.8. Antioxidant Activity of the Prepared Croissants

To verify the maintenance of the antioxidant activity measured in elderberry juice (Section 3.3.1) in the final products, the same assays (DPPH radical-scavenging, reducing power, and inhibition of β-carotene bleaching) were tested in selected croissant formulations. Extracts were prepared from 3 g of lyophilised croissant with 30 mL of ethanol:water (80:20 *v*/*v*); successive dilutions were further prepared from each extract.

### 3.9. Statistical Analysis

All assays were performed in triplicate and the results are expressed as mean values±standard deviation (SD). Results were compared through analysis of variance (ANOVA) using Tamhane’s T2 test (α = 0.05), since all sample distributions turned out to be heteroscedastic, as verified through the the Levene’s test to verify the homogeneity of variances (Levene’s test). Statistical tests were done with SPSS v. 23.0 (IBM Corp., Armonk, NY, USA).

## 4. Conclusions

All anthocyanins characterised in *S. nigra* juice derive from cyanidin, presenting cyanidin-3-*O*-sambubioside and cyanindin-3-*O*-glucoside as the major compounds.

From a nutritional point of view, the prepared croissants did not present a very significant variation between different formulations. Nevertheless, some significant differences were found among free sugars and fatty acids profiles. Even so, the highest differences were detected in the appearance of the final products (particularly regarding a* values) and their antioxidant activity. When considering all results together, it might be concluded that the incorporation of 4% of elderberry juice was the most suitable solution, either considering the improvement in croissants’ appearance, antioxidant activity, as well as the need to guarantee dietary values of anthocyanins below the threshold value that is typically defined for this type of phenolic compounds (50 mg/day). In addition, elderberry juice provided the same benefits as those achieved with the incorporation of black carrot commercial dye, raising the potential of elderberries to be used at industrial level.

## Figures and Tables

**Table 1:** Bioactivity and anthocyanin profile of *Sambucus nigra* juice.

**Antioxidant Activity (EC_50_, mg/mL)**					
DPPH Radical-Scavenging			Reducing Power		β-Carotene Bleaching Inhibition
3.1 ± 0.1			3.7 ± 0.2		9.4 ± 0.3
**Antitumor Activity (GI_50_, μg/mL)**					
MCF-7		HeLa	NCI-H460	HepG2	PLP2
58 ± 1		16 ± 1	16 ± 1	98 ± 4	>400
**Anthocyanins Profile of *S. nigra***					
Peak	RT (min)	λ_max_ (nm)	Pseudomolecular ion [M + H]^+^ (*m/z*)	Identification	Quantification (μg/mL)
1	25.31	515	581(79), 449(100), 287(77)	Cyanidin-3-*O*-sambubioside-5-*O*-glucoside	140 ± 21
2	36.63	517	287(100)	Cyanidin-3-*O*-sambubioside	560 ± 45
3	38.26	517	287(100)	Cyanidin-3-*O*-glucoside	390 ± 30
				Total anthocyanins	1090± 35

EC_50_/GI_50_: concentration corresponding to 50% of activity; MCF-7: human breast adenocarcinoma; HeLa: human cervical carcinoma; NCI-H460: human non-small lung carcinoma; HepG2: human hepatocellular carcinoma; PLP2: non-tumour primary culture of porcine liver cells.

**Table 2:** Nutritional value and free sugars identified in the different croissant formulations.

Sample	Nutritional Value (g/100 g)					Energy (Kcal/100 g)	Free Sugars (g/100 g)				
	Moisture	Fat	Proteins	Carbohydrates	Ash		Fructose	Glucose	Maltose	Trehalose	Sugars
BC	0.23 ± 0.02	17.0 ± 0.4 a	10.3 ± 0.2 b	70 ± 1	2.0 ± 0.1	474 ± 2 ab	4.1 ± 0.1 b	2.4 ± 0.1 b	3.9 ± 0.2 a	1.8 ± 0.1 a	12.2 ± 0.2 b
EJ2	0.23 ± 0.03	16.7 ± 0.3 b	10.4 ± 0.3 b	71 ± 1	2.1 ± 0.1	476 ± 4 a	5.1 ± 0.3 a	2.4 ± 0.3 b	3.7 ± 0.1 b	1.6 ± 0.1 b	12.8 ± 0.5 ab
EJ4	0.24 ± 0.02	16.4 ± 0.4 bc	11.0 ± 0.1 a	70 ± 1	2.1 ± 0.2	472 ± 4 b	5.2 ± 0.5 a	2.7 ± 0.5 b	3.6 ± 0.1 b	1.4 ± 0.1 c	12.9 ± 0.5 ab
EJ8	0.24 ± 0.03	16.1 ± 0.2 c	11.1 ± 0.1 a	71 ± 1	2.0 ± 0.1	473 ± 2 b	5.6 ± 0.5 a	3.4 ± 0.5 a	4.0 ± 0.1 a	1.3 ± 0.2 cd	14,3 ± 0.5 a
CCD	0.24 ± 0.02	16.5 ± 0.2 b	10.9 ± 0.1 a	70 ± 1	2.0 ± 0.1	472 ± 2 b	4.2 ± 0.2 b	1.6 ± 0.1 c	3.6 ± 0.1 b	1.2 ± 0.2 d	10.6 ± 0.2 c
Homoscedasticity ^1^ (*p* value) (*n* = 45)	<0.001	0.006	<0.001	0.012	<0.001	0.001	<0.001	<0.001	<0.001	0.003	<0.001
ANOVA ^2^ (*p* value) (*n* = 45)	0.191	0.001	<0.001	0.071	0.084	<0.001	<0.001	<0.001	<0.001	<0.001	<0.001

^1^*p* values below 0.05 indicate heteroscedastic distributions, and the multiple comparison was made by the Tamhane’s T2 test. ^2^ As the value of *p* is below 0.05 in all cases; the corresponding parameters present significant differences for at least one of the croissant formulations (identified with different letters). BC: croissant with no colourants adding; EJ2: croissant with 2% of elderberry juice (EJ2); EJ4: croissant with 4% of elderberry juice; EJ8: croissant with 8% of elderberry juice; CCD: croissant with 4% of the commercial carrot dye.

**Table 3:** Fatty acid profiles of the different croissant formulations (relative percentage).

	C6:0	C10:0	C12:0	C14:0	C16:0	C16:1	C18:0	C18:1	C18:2	C18:3n3	SFA	MUFA	PUFA
BC	0.9 ± 0.1 c	1.4 ± 0.1 c	1.9 ± 0.1 c	5.9 ± 0.1 c	21 ± 1 b	1.0 ± 0.1 d	7.0 ± 0.1 b	28 ± 1 cd	28 ± 1 a	0.7 ± 0.1	41 ± 1 c	30 ± 1 c	29 ± 1 b
EJ2	1.0 ± 0.2 c	1.3 ± 0.1 c	1.7 ± 0.3 c	4.7 ± 0.5 d	17 ± 1 c	1.1 ± 0.2 cd	6.8 ± 0.4 b	28 ± 1 d	29 ± 2 a	1.3 ± 0.1	37 ± 2 d	30 ± 1 bc	33 ± 3 a
EJ4	1.3 ± 0.1 b	1.7 ± 0.2 b	2.2 ± 0.2 b	6.3 ± 0.4 bc	19 ± 1 c	1.4 ± 0.1 ab	8.0 ± 0.1 a	30 ± 1 a	22 ± 1 b	1.3 ± 0.1	44 ± 1 b	32 ± 1 a	24 ± 1 c
EJ8	1.6 ± 0.1 a	1.9 ± 0.1 a	2.5 ± 0.1 a	7.6 ± 0.1 a	24 ± 3 a	1.3 ± 0.1 bc	8.3 ± 0.5 a	29 ± 1 bc	20 ± 2 b	1.0 ± 0.1	47 ± 2 a	31 ± 1 b	22 ± 2 c
CCD	1.0 ± 0.1 c	1.8 ± 0.2 ab	2.2 ± 0.2 b	6.8 ± 0.5 ab	21 ± 1 b	1.6 ± 0.2 a	8.3 ± 0.4 a	29 ± 1 ab	22 ± 2 b	0.9 ± 0.1	44 ± 1 b	32 ± 1 a	24 ± 2 c
Homoscedasticity ^1^ (*p* value) (*n* = 45)	<0.001	0.003	<0.001	<0.001	<0.001	<0.001	<0.001	<0.001	<0.001	<0.001	<0.001	<0.001	0.001
ANOVA^2^ (*p* value) (*n* = 45)	<0.001	<0.001	<0.001	<0.001	<0.001	0.017	<0.001	<0.001	0.042	0.072	0.041	0.006	<0.001

C6:0—Caproic acid, C:10—Decanoic acid, C12:0—Lauric acid, C14:0—Myristic acid, C16:0—Palmitic acid, C18:0—Stearic acid, C18:1—Oleic acid, C18:2—Linoleic acid, C18:3n3—α-linolenic acid, SFA: saturated fatty acids, MUFA: monounsaturated fatty acids, PUFA—polyunsaturated fatty acids. ^1^ *p* values below 0.05 indicate heteroscedastic distributions, and the multiple comparisons were made by the Tamhane’s T2 test. ^2^ As the value of *p* is below 0.05 in all cases; the corresponding parameters present significant differences for at least one of the croissant formulations (identified with different letters). BC: croissant with no colourants adding; EJ2: croissant with 2% of elderberry juice (EJ2); EJ4: croissant with 4% of elderberry juice; EJ8: croissant with 8% of elderberry juice; CCD: croissant with 4% of the commercial carrot dye.

**Table 4:** Colour parameters (L*, a* e b*) of different croissant formulations.

	L*	a*	b*
BC	79 ± 1 a	6.4 ± 0.1 c	31 ± 1 a
EJ2	62 ± 1 c	8.5 ± 0.4 b	19 ± 1 b
EJ4	57 ± 1 d	13.9 ± 0.1a	12 ± 1c
EJ8	54 ± 1 e	14.0 ± 0.1a	9 ± 1d
CCD	69 ± 3 b	8.6 ± 0.1 b	19 ± 1 b
Homoscedasticity ^1^ (*p*-value) (*n* = 45)	<0.001	<0.001	<0.001
ANOVA ^2^ (*p*-value) (*n* = 45)	<0.001	<0.001	<0.001

^1^*p*-values below 0.05 indicate heteroscedastic distributions, and the multiple comparison was made by the Tamhane’s T2 test. ^2^ As the value of *p* is less than 0.05 in all cases, the corresponding parameters present significant differences for at least one croissant formulation (identified with different letters). BC: croissant with no colourants adding; EJ2: croissant with 2% of elderberry juice (EJ2); EJ4: croissant with 4% of elderberry juice; EJ8: croissant with 8% of elderberry juice; CCD: croissant with 4% of the commercial carrot dye.
